# Supplementary material for: The virulent bacteriophage Henu8 as an antimicrobial synergist against Escherichia coli
Source: Microbiol Spectr. 2025 May 16;13(7):e01633-24. doi: 10.1128/spectrum.01633-24 (PMC12210894; doi:10.1128/spectrum.01633-24)
Supplement: Figure S1 to S4 and Table S1 — Clinical isolate data. [file spectrum.01633-24-s0001.doc]

**The virulent bacteriophage Henu8 as an antimicrobial synergist against *Escherichia coli***

Fang Zhou^†1^, Kexiao Wang^†1^, Shuai Ji^2^, Xiaochen Liao^2^, Wenwen Zhang^1^, Tieshan Teng^1,3^, Li Wang*^1^, Qiming Li*^1,2,3^

*^1^**Department of Clinical Laboratory, The First Affiliated Hospital of Henan University, Henan University, Kaifeng 475000, China.*

*^2^The Jointed National Laboratory of Antibody Drug Engineering, Henan University, Kaifeng 475000, China.*

*^3^Department of Microbiology, College of Basic Medical Sciences, Henan Medical School, Henan University, Kaifeng 475000, China.*

^*^Corresponding author. E-mail: wangli851217@163.com; liqiming82@126.com

^†^These authors contributed equally to this work.


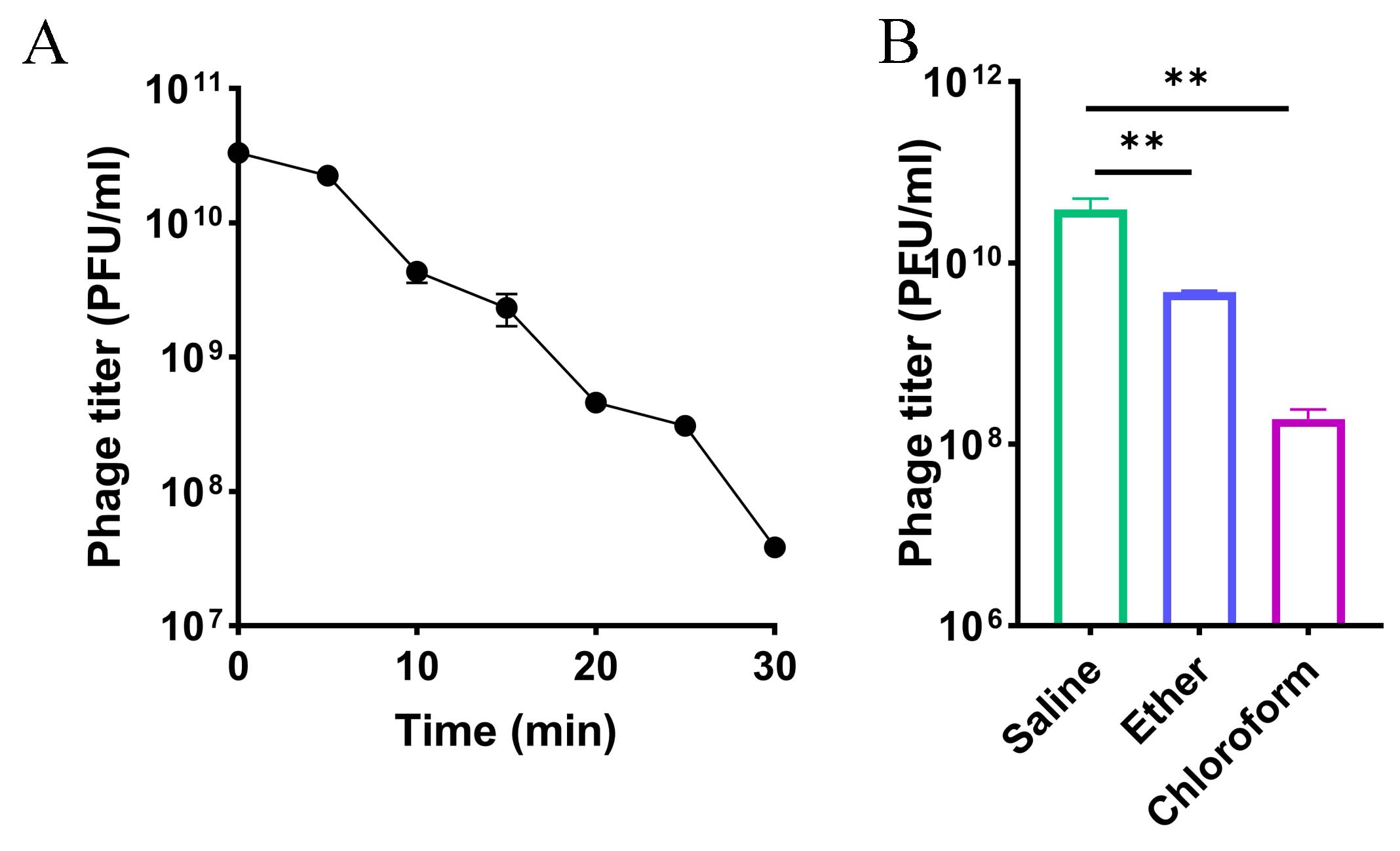


**Fig. S1.** (A) UV stability of the bacteriophage Henu8. PFU was detected every 5 min (up to 30 min) under UV irradiation. (B) Stability in organic solvents. Chloroform and ether were mixed with the bacteriophage Henu8. After 60 min of incubation, the PFU was determined via the plaque counting method. The data are presented as the means ± standard deviations. Statistical analysis was performed via one-way analysis of variance following Dunnett's multiple comparisons test. **P < 0.01.


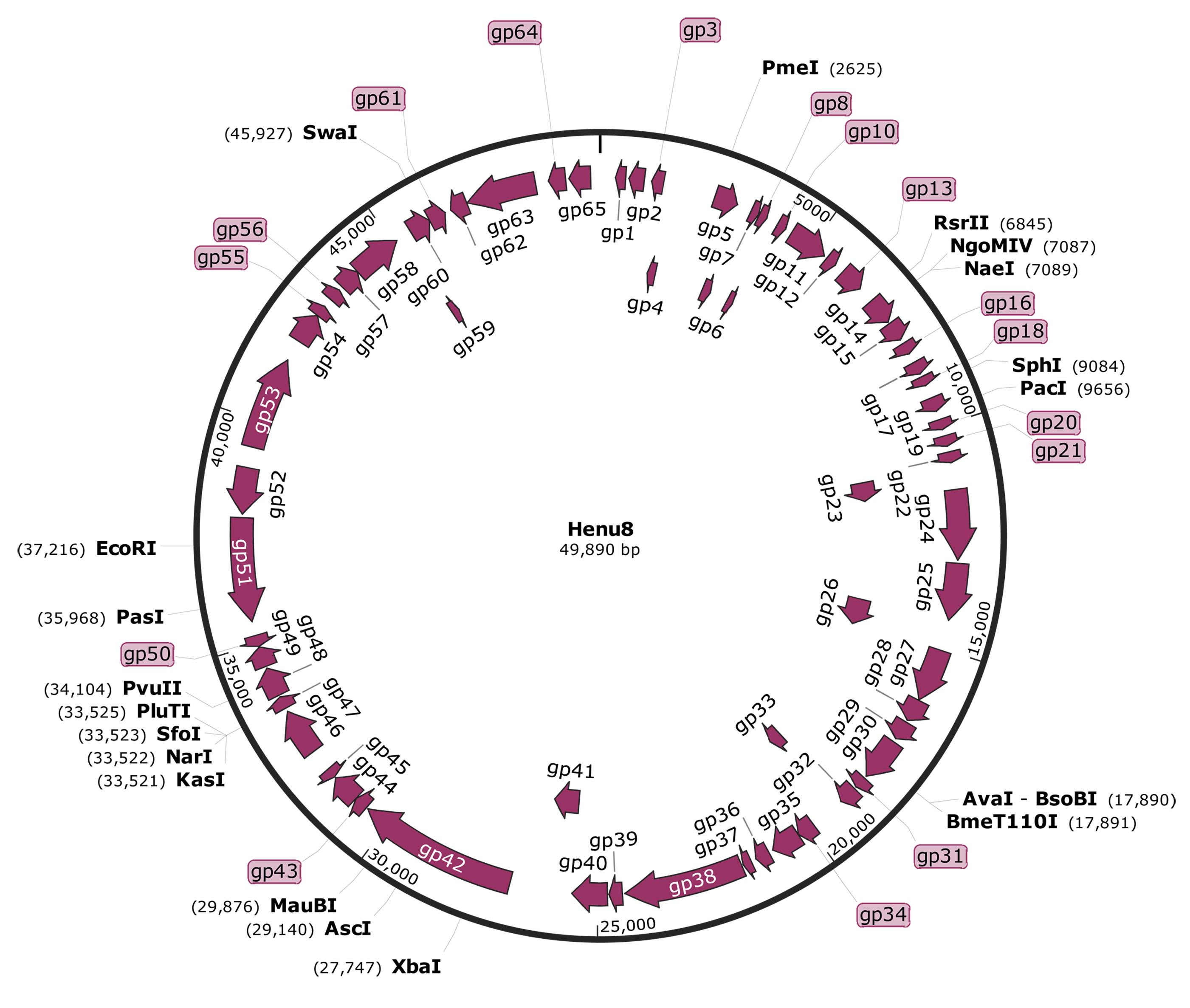


**Fig. S2.** The ORFs and restriction site of the bacteriophage Henu8.


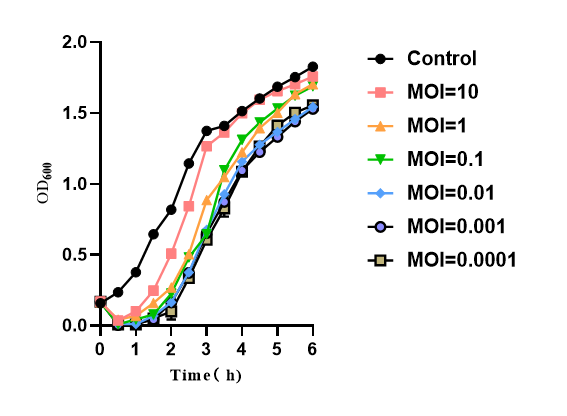


**Fig. S3.** *In vitro* evaluation of lytic efficacy against *E. coli* A57. The bacteriophage Henu8 was coincubated with *E. coli* A57 at the indicated MOIs in a shaking incubator, where samples were taken every 30 min for absorbance tests at 600 nm.


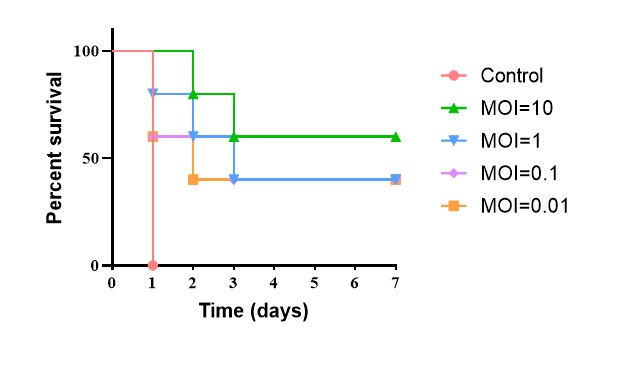


**Fig. S4.** Phage therapy of bacteriophage Henu8 in a C57BL/6 N mouse *E. coli* A57 bacteremia model. Mice infected with *E. coli* A57 at a CFU/mL of 5×10^9^ received phage therapy at different MOIs (10, 1, 0.1, and 0.01), the survival of mice were observed and recorded for 7 days.

Table S1. MICs of antibiotics against *E. coli* A57

| Antibiotics | MIC (μg/ml) |
| --- | --- |
|  | Met |
| Ampicillin | ≧32 |
| Gentamicin | ≧16 |
| Cefazolin | ≧8 |
| Chloramphenicol | ≧32 |
| Ceftazidime | ≧32 |
| Minocycline | ≦4 |
| Ticacillin/clavic acid | ≧128/2 |
| Cefoperazone/Sulbactam | ≧64/32 |
| Ciprofloxacin | ≧4 |
| Levofloxacin | ≧8 |
| Trimesulf | ≧8/152 |
| Amikacin | ≦4 |
| Imipenem | ≦1 |
| Meropenem | ≦1 |
| Ampicillin/sulbactam | ≧64/32 |
| Piperacillin/Tazobactam | ≦4/4 |
| Ceftriaxone | ≧64 |
| Cefoxitin | ≦8 |
| Cefepime | ≧32 |
| Cefuroxime | ≧32 |
